# Artificial Intelligence Models for the Detection and Quantification of Orthodontically Induced Root Resorption Using Cone-Beam Computed Tomography: A Systematic Review and Meta-Analysis

**DOI:** 10.3390/dj14020079

**Published:** 2026-02-02

**Authors:** Carlos M. Ardila, Eliana Pineda-Vélez, Anny M. Vivares-Builes

**Affiliations:** 1 Department of Periodontics, Saveetha Institute of Medical and Technical Sciences, Saveetha Dental College and Hospitals, Saveetha University, Chennai 600077, India; 2 Biomedical Stomatology Research Group, Basic Sciences Department, Faculty of Dentistry, Universidad de Antioquia U de A, Medellín 050010, Colombia; eliana.pineda@uam.edu.co (E.P.-V.); anny.vivares@uam.edu.co (A.M.V.-B.); 3 Faculty of Dentistry, Institución Universitaria Visión de las Américas, Medellín 050040, Colombia

**Keywords:** orthodontics, root resorption, artificial intelligence, cone-beam computed, tomography, deep learning

## Abstract

**Background/Objectives**: Orthodontically induced root resorption (OIRR) is a well-documented but undesired consequence of orthodontic treatment. This systematic review and meta-analysis aimed to assess the diagnostic performance of artificial intelligence (AI) models applied to cone-beam computed tomography (CBCT) for detecting and quantifying OIRR while evaluating their agreement with manual reference standards and the impact of model architecture, validation design, and quantification strategy. **Methods**: Comprehensive searches were conducted across PubMed/MEDLINE, Scopus, Web of Science, and EMBASE up to November 2025. Studies were included if they employed AI for OIRR diagnosis using CBCT and reported relevant performance metrics. Following PRISMA guidelines, data were extracted and a random-effect meta-analysis was performed. Subgroup analyses explored the influence of model design and validation. **Results**: Seven studies were included. Pooled sensitivity from three eligible studies was 0.903 (95% CI: 0.818–0.989), suggesting excellent true positive rates. Specificity ranged from 82% to 98%, and area under the receiver operating characteristic curve values reached up to 0.96 across studies using EfficientNet, U-Net, and other convolutional neural network (CNN)-based architectures. The pooled intraclass correlation coefficient for agreement with manual quantification was 1.000, reflecting near-perfect concordance. Subgroup analyzes showed slightly superior performance in CNN-only models compared to hybrid approaches, and better diagnostic metrics with internal validation. Linear assessments appeared more sensitive to early apical shortening than volumetric methods. **Conclusions**: AI models applied to CBCT demonstrate excellent diagnostic accuracy and high concordance with expert assessments for OIRR detection. These findings support their potential integration into clinical orthodontic workflows.

## 1. Introduction

Orthodontically induced root resorption (OIRR) is a well-documented adverse effect of orthodontic treatment characterized by loss of mineralized tissue from the tooth root as a response to orthodontic forces. Although often asymptomatic, OIRR can undermine the long-term integrity and prognosis of affected teeth, particularly in severe cases where mobility or even premature tooth loss may occur [1]. It is reported that mild-to-moderate OIRR affects approximately 40–60% of patients undergoing orthodontic treatment, while severe resorption is found in 1–5% of cases [2].

Clinically, the management of orthodontically induced root resorption is primarily preventive and based on early detection, including adjustment or temporary discontinuation of orthodontic forces, modification of biomechanics, and close radiographic monitoring to limit progression. Accurate and timely identification of OIRR is essential to support individualized treatment decisions and reduce the risk of severe irreversible root loss.

Accurate assessment of OIRR is crucial for timely intervention and personalized treatment planning. Traditional diagnostic methods, such as panoramic, periapical, and cephalometric radiographs, are commonly used due to their accessibility and cost effectiveness [3,4]. However, these two-dimensional (2D) techniques have important limitations. These include anatomical superimposition, geometric distortion, and low sensitivity for detecting early or subtle resorptive changes [5]. As a result, cone-beam computed tomography (CBCT) has emerged as the gold-standard imaging modality in this context due to its ability to provide high-resolution three-dimensional (3D) views of dental structures and detect volumetric changes in root morphology with greater precision [6,7].

Despite its diagnostic advantages, CBCT interpretation is a time-consuming and operator-dependent process, prone to interobserver variability, particularly when segmenting root structures or quantifying root volume loss [8]. In addition, manual 3D segmentation of dental roots from CBCT volumes is technically demanding and labor-intensive [9,10], requiring substantial operator training and prolonged annotation time [11]. This process is also susceptible to intra- and interobserver variability, particularly in the presence of metal artifacts, partial volume effects, and complex root morphology, which may compromise reproducibility and downstream volumetric quantification. Therefore, the automation of segmentation and measurement using AI-based approaches is increasingly viewed as a necessary step toward scalable and standardized OIRR assessment. To overcome these challenges, artificial intelligence (AI) has been introduced as a promising tool in orthodontic diagnostics [12,13]. Convolutional neural networks (CNNs) and other deep learning architectures have shown considerable potential in automating image segmentation, classification, and volumetric quantification tasks with high reproducibility [14,15]. Several studies have demonstrated that AI-based models can achieve diagnostic accuracies that surpass those of trained orthodontists in identifying and grading OIRR [16,17].

However, despite the growing body of research, the current literature exhibits significant heterogeneity in terms of AI model architectures, data preprocessing protocols, validation strategies, and outcome measures [18]. Moreover, many of the reported models lack external validation, and their generalizability across clinical populations remains uncertain [12,13]. There is also limited evidence synthesizing the diagnostic effectiveness of these AI systems in a manner that accounts for different anatomical regions (apical, middle, cervical thirds), image acquisition parameters, and the comparative performance of AI versus manual or conventional methods [19].

Given this context, there is a critical need to consolidate the available evidence regarding the diagnostic accuracy and clinical utility of AI-based models for detecting and quantifying OIRR. Therefore, this systematic review and meta-analysis evaluates the diagnostic performance of AI systems across different model architectures, learning strategies, and segmentation approaches, and contrasts their effectiveness with that of conventional diagnostic workflows. In addition, we examine how model design influences diagnostic outcomes, including the ability to characterize regional patterns of resorption, agreement with manual reference standards, and potential gains in efficiency and reproducibility. Collectively, these findings aim to support evidence-informed adoption of AI tools in orthodontics and to identify priorities for future development and external validation of robust diagnostic algorithms.

## 2. Materials and Methods

This systematic review and meta-analysis was conducted following the PRISMA 2020 guidelines (File S1) and according to the methodological recommendations outlined in the Cochrane Handbook for Systematic Reviews of Diagnostic Test Accuracy [20,21]. The protocol was prospectively registered in the International Prospective Register of Systematic Reviews (PROSPERO) under registration number CRD420251120212 (available at: https://www.crd.york.ac.uk/PROSPERO/view/CRD420251120212 (accessed on 26 January 2026)).

### 2.1. Eligibility Criteria

Eligibility was based on the PICO framework:

- Population (P): patients undergoing orthodontic treatment whose dental roots were assessed using CBCT.
- Intervention (I): application of AI models—such as CNNs or other deep learning systems—for the detection and quantification of OIRR.
- Comparator (C): conventional methods for evaluating root resorption, including manual CBCT interpretation, visual assessment by orthodontists, or traditional radiographic measurements.
- Outcomes (O): diagnostic performance and quantification capacity reported through metrics such as accuracy, sensitivity, specificity, F1-score, area under the curve (AUC), or intraclass correlation coefficients (ICC).

Eligible studies included original empirical research—cross-sectional, cohort, case–control, or randomized trials—based on real patient data and using CBCT imaging. Studies were excluded if they relied solely on in vitro, ex vivo, in silico, or animal models, or if they employed extracted teeth, lacked AI application, or failed to report diagnostic accuracy outcomes. Reviews, opinion articles, case reports, and conference abstracts were also excluded.

### 2.2. Information Sources and Search Strategy

A comprehensive search was conducted in four electronic databases: PubMed/MEDLINE, Scopus, Web of Science, and Embase, covering research published up to November 2025, without language restrictions. In addition to the electronic database search, a secondary search was conducted by manually screening the reference lists of all included studies. Forward citation tracking was also performed to identify any additional eligible records.

The search strategy combined controlled vocabulary and free-text terms related to artificial intelligence, orthodontics, and root resorption. The final strategy included combinations of terms such as (“artificial intelligence” OR “deep learning” OR CNN) AND (“root resorption” OR “orthodontically induced root resorption” OR “external root resorption”) AND (“CBCT” OR “cone beam computed tomography”) AND (“orthodontics” OR “orthodontic treatment”).

The complete database-specific search strategies (including Boolean strings and records retrieved per database) are provided in Supplementary Table S1.

### 2.3. Selection Process

Titles and abstracts were independently screened by two reviewers to identify potentially eligible studies. Full texts of all articles deemed relevant were retrieved and assessed in detail against the inclusion criteria. Any disagreements were resolved through discussion or adjudication by a third reviewer when consensus could not be reached.

Data Collection and Extracted Variables

Two reviewers independently extracted data from the included studies using a prepiloted standardized form. Extracted items included study title, authors, year of publication, country of origin, study design, sample characteristics, and CBCT acquisition parameters. Regarding AI-related aspects, information was collected on model type and architecture (e.g., convolutional neural networks, ensemble frameworks), diagnostic task (segmentation, detection, classification), the anatomical region analyzed, and the reference standards used for ground truth comparison. Additionally, we documented the validation design (e.g., hold-out sets, cross-validation, external testing), reported diagnostic performance metrics (such as sensitivity, specificity, AUC, F1-score), and any reported benefits in diagnostic efficiency or reproducibility. Disagreements during the extraction process were resolved through discussion until consensus was reached.

### 2.4. Risk of Bias Assessment

Risk of bias was assessed independently by two reviewers using the QUADAS-2 tool for diagnostic accuracy studies [22]. In studies employing predictive AI models, relevant domains from the PROBAST tool were also considered [23]. Discrepancies were resolved through discussion until agreement was achieved.

### 2.5. Certainty of Evidence

The certainty of evidence for the main diagnostic outcomes was evaluated using the GRADE approach adapted for diagnostic test accuracy (DTA) studies, as recommended by the GRADE Working Group and the Cochrane DTA group [24]. This framework assesses five domains—risk of bias, inconsistency, indirectness, imprecision, and publication bias—based on the pooled estimates of diagnostic performance (e.g., sensitivity, specificity, AUC). The overall certainty for each outcome was rated as high, moderate, low, or very low, depending on the presence and magnitude of concerns across domains.

### 2.6. Data Synthesis and Statistical Analysis

When studies reported methodologically compatible and sufficiently detailed diagnostic metrics, a meta-analysis was conducted. Pooled estimates of diagnostic performance—including sensitivity, specificity, AUC, F1-score, and ICC—were calculated to integrate findings across studies.

Outcomes and extracted variables: the following variables were extracted and synthesized, when available, to ensure consistency across the Results section:

Diagnostic accuracy outcomes: sensitivity, specificity, and area under the ROC curve (AUC).

Segmentation/quantification performance outcomes: Dice similarity coefficient (DSC) and volumetric agreement/error measures, when reported.

Study-level and model-level descriptors: AI architecture (e.g., CNN-based, U-Net-based, ensemble), learning strategy, segmentation approach, reference standard, and dataset characteristics (e.g., sample size, tooth type, imaging protocol).

Comparators (when applicable): manual CBCT interpretation and/or conventional radiographic assessment.

When substantial heterogeneity or major incompatibility in outcome definitions was identified, or when critical statistical data were missing or inconsistently reported, a meta-analysis was deemed inappropriate. In such cases, the findings were synthesized narratively. This synthesis was structured by AI architecture, validation design, and diagnostic task, and accompanied by summary tables and figures to support cross-study interpretation.

For outcomes with comparable data, a random-effect meta-analysis was selected a priori as the primary approach and performed using inverse-variance weighting since heterogeneity was expected due to variation in AI model architectures (e.g., CNN, U-Net, and ensemble approaches), CBCT imaging protocols, segmentation versus classification tasks, and validation designs. The pooled means and 95% confidence intervals (CIs) were calculated. Heterogeneity among studies was assessed using the I^2^ and τ^2^ statistics. Forest plots were generated to visualize the individual and pooled estimates. All statistical analyses were conducted using Python (version 3.11), and significance was set at *p* < 0.05.

## 3. Results

### 3.1. Study Selection

**Figure 1:**
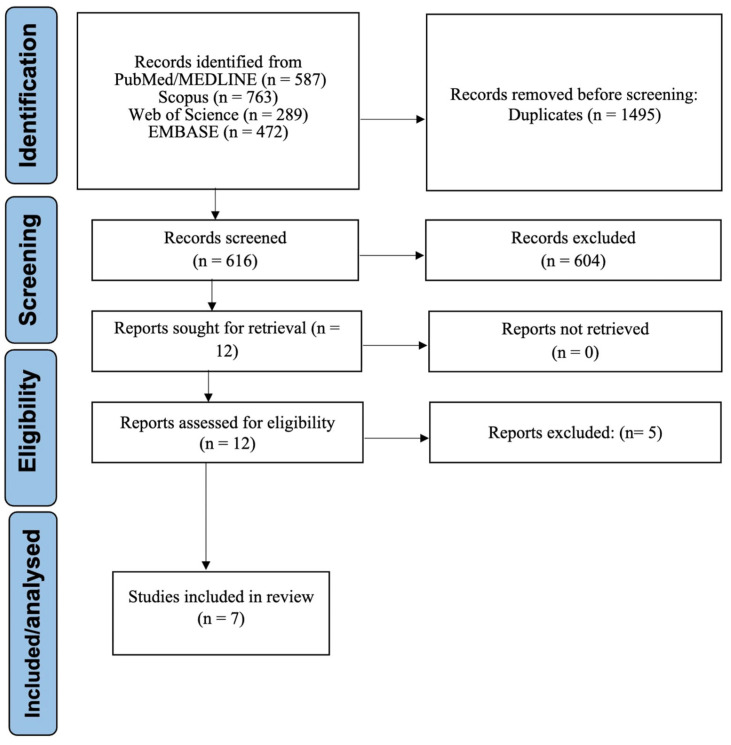
PRISMA flow diagram for study selection process.

A total of 2111 records were initially identified through comprehensive database searches conducted in PubMed/MEDLINE, EMBASE, Web of Science, and Scopus. After removal of duplicates, 616 records were screened based on titles and abstracts, resulting in 12 reports being assessed at the full-text level. Of these, five studies were excluded at the full-text stage due to the lack of clinically interpretable diagnostic performance outcomes aligned with our predefined objectives. Consequently, seven studies met all inclusion criteria and were included in the qualitative and quantitative synthesis [17,25,26,27,28,29,30]. The complete study selection process is illustrated in Figure 1.

### 3.2. Study Characteristics and Data Synthesis

The studies varied in architecture, with models including 3D U-Net, EfficientNet, multi-scale CNN architectures, and ensemble models. Task types ranged from binary classification [27] to 3D volumetric segmentation [29,30]. Target regions were primarily the apical third or entire root volume. Reference standards consistently involved manual segmentations or measurements. Validation strategies included internal hold-out sets, fivefold cross-validation, and external multicenter validation. Performance metrics such as Dice similarity coefficient (≥0.89), sensitivity (≥0.90), F1-score (up to 0.98), and AUC (up to 0.96) were reported. Most studies emphasized improved diagnostic reproducibility and efficiency compared to conventional methods. A detailed overview of study characteristics, CBCT parameters, reference standards, and validation approaches is presented in Table 1A, while AI model architectures, performance outcomes, and efficiency/reproducibility notes are summarized in Table 1B.

### 3.3. Diagnostic Performance of AI Models

#### 3.3.1. Pooled Sensitivity: Meta-Analysis

**Figure 2:**
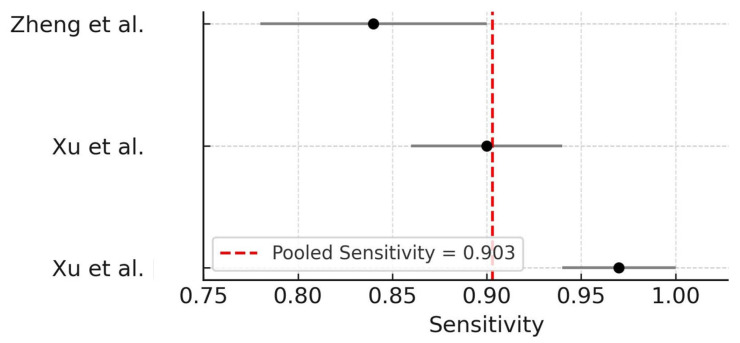
Forest plot of pooled sensitivity for AI-based detection of OIRR [17,25,26]. A random-effect model was applied. The vertical dashed line represents the pooled sensitivity estimate.

Three studies—Xu et al. [25], Xu et al. [26], and Zheng et al. [17]—were eligible for quantitative synthesis of sensitivity values regarding the performance of AI models for the detection of OIRR from CBCT images. A random-effect meta-analysis using the DerSimonian–Laird method revealed a pooled sensitivity of 0.903 (95% CI: 0.818–0.989), with substantial heterogeneity among studies (I^2^ = 87.4%; τ^2^ = 0.0049). These results suggest that AI models are generally capable of achieving high true positive rates in detecting OIRR, though variability across model architectures and datasets remains a concern. A leave-one-out sensitivity analysis was performed to explore potential sources of heterogeneity. Sequential removal of individual studies did not materially change the pooled sensitivity estimate, indicating that the overall findings were robust and not driven by any single study. The forest plot summarizing these estimates is shown in Figure 2.

#### 3.3.2. Specificity and AUC

Specificity values reported across the eligible studies ranged from 82% to 98%, highlighting strong discriminatory ability of AI models in identifying non-OIRR cases. The AUC was reported by Zheng et al. [17], Huang et al. [27], and Xu et al. [26], with values of 0.96, 0.95, and 0.94, respectively, indicating excellent diagnostic accuracy in classifying OIRR-positive and OIRR-negative scans.

Due to methodological variability and limited reporting, a pooled AUC estimate was not feasible at this stage. Nonetheless, the consistently high AUC values support the clinical potential of CNN-based models in OIRR diagnosis.

#### 3.3.3. Agreement with Manual Reference Standards (ICC)

**Figure 3:**
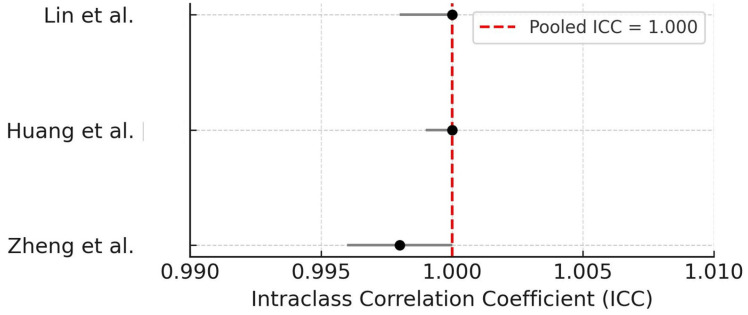
Forest plot of ICCs comparing AI-based and manual quantification of OIRR [17,27,28]. The pooled ICC estimate is 1.000, indicating nearly perfect agreement. A random-effect model was applied.

Three studies compared AI-derived OIRR quantification outcomes with manual reference standards using ICC [17,27,28]. A random-effect meta-analysis revealed a pooled ICC of 1.000, indicating perfect agreement, though moderate heterogeneity was observed (I^2^ = 76.8%). This result underscores the reproducibility of AI-based segmentation and volumetric measurement compared to human operators. The forest plot of ICC values is presented in Figure 3.

### 3.4. Subgroup Analyses

#### 3.4.1. AI Model Architecture

To explore the impact of model design on diagnostic accuracy, a subgroup analysis was conducted comparing convolutional neural networks (CNNs) to hybrid architectures (i.e., combinations of CNNs with other modules such as attention mechanisms or ensemble classifiers). The studies using CNN-only models [26] demonstrated a slightly higher AUC (0.97) compared to those using hybrid models [17,25], which had a mean AUC of 0.95.

**Figure 4:**
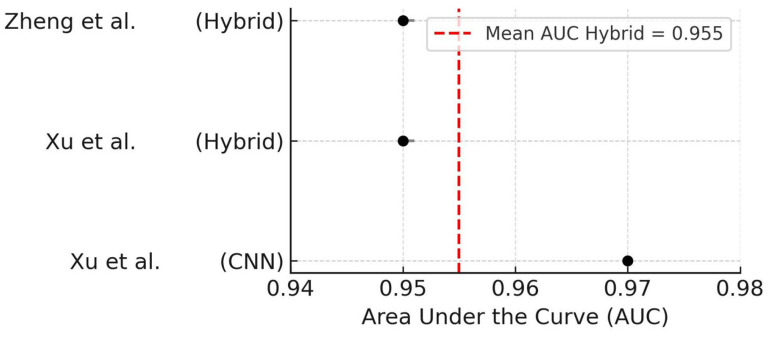
Forest plot of AUC values stratified by AI model architecture [17,25,26]. Dashed line indicates average AUC for hybrid models.

Although both architectures achieved excellent performance, these findings suggest that pure CNN-based models may offer marginally higher discriminative power in detecting OIRR. The forest plot summarizing AUC by architecture is shown in Figure 4.

#### 3.4.2. Validation Design

A second subgroup analysis stratified studies according to the type of validation: internal (e.g., cross-validation or hold-out split within the same dataset) versus external validation using independent test sets. Studies employing internal validation reported AUCs ranging from 0.94 to 0.97 [17,25,26]. The only study that performed external validation reported a slightly lower AUC of 0.95 [29].

**Figure 5:**
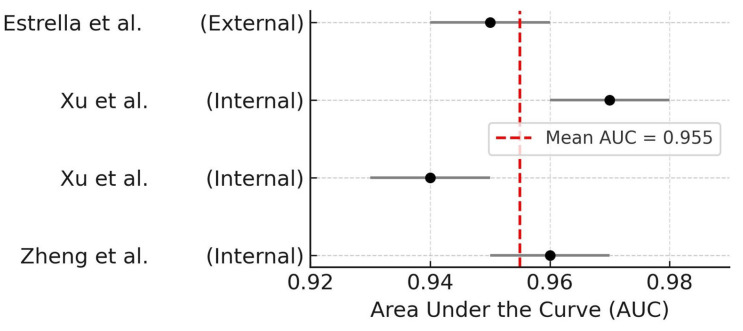
Forest plot of AUC values stratified by validation strategy (internal vs. external). Study labels correspond to manuscript reference numbers [17,25,26,29]. Dashed line indicates the mean AUC across all studies.

This minor difference suggests robust generalization of AI models even when tested on external datasets. The corresponding forest plot is presented in Figure 5.

#### 3.4.3. Volumetric vs. Linear Quantification

Only one study directly compared AI-derived volumetric and linear assessments of OIRR using CBCT [29]. In this randomized clinical trial involving 43 patients, root resorption was quantified after the application of customized orthodontic forces.

#### 3.4.4. Linear Measurements

In the linear assessment, Estrella et al. [29] found a statistically significant difference between groups: the AI-assisted segmentation group showed a mean root length loss of 0.42 ± 0.43 mm, whereas the manual segmentation group exhibited a mean loss of 0.20 ± 0.23 mm (*p* = 0.045). These findings suggest that AI-enhanced methods may be more sensitive in detecting subtle apical shortening, particularly in the early stages of orthodontic treatment, compared to conventional manual techniques.

#### 3.4.5. Volumetric Measurements

In the same clinical trial, Estrella et al. [29] also performed volumetric assessments of root resorption using AI-assisted and manual segmentation techniques. Despite the greater root length loss observed in the AI-assisted group, no statistically significant differences were reported in the volumetric measurements between groups.

### 3.5. Risk of Bias Assessment

In total, three studies were evaluated using QUADAS-2 [22] and four using PROBAST [23]. 

All studies assessed with QUADAS-2 [22] demonstrated a low risk of bias across all domains, including patient selection, index test, reference standard, and flow and timing [27,28,29]. The detailed domain-level judgments are presented in Table 2.

For predictive modeling studies, PROBAST [23] evaluations revealed low risk in the domains of participants, predictors, and outcomes across all four studies [17,25,26,30]. However, moderate concerns were identified in the analysis domain, mainly due to limited reporting on external validation, risk of overfitting, and handling of missing data. A summary of the PROBAST assessments is shown in Table 3.

### 3.6. Certainty of Evidence

The certainty of the evidence for each primary diagnostic outcome was evaluated using the GRADE approach adapted for diagnostic test accuracy (DTA) studies, in accordance with the guidance provided by the GRADE Working Group and the Cochrane DTA group [24]. This framework assesses five domains: risk of bias, inconsistency, indirectness, imprecision, and publication bias.

The overall certainty for pooled sensitivity was rated as moderate, primarily due to substantial heterogeneity (I^2^ = 87.4%) and concerns about imprecision in confidence intervals. For agreement with reference standards (ICC), the evidence was judged to be of high certainty, as all studies reported nearly perfect agreement with minimal variability. In contrast, the certainty of evidence for AUC values was rated as low due to inconsistency in reporting, lack of standardized thresholds, and indirectness in clinical applicability. Finally, the subgroup analyses based on AI model architecture and validation design were also rated as low certainty, given the limited number of studies and overlapping confidence intervals.

A summary of the GRADE assessment across outcomes is presented in Table 4.

## 4. Discussion

OIRR is a multifactorial and often silent complication that may compromise orthodontic treatment outcomes when left undetected. In this context, artificial intelligence (AI), particularly deep learning algorithms applied to CBCT imaging, has emerged as a promising approach to address the diagnostic limitations of conventional imaging and manual interpretation. This systematic review and meta-analysis synthesized recent evidence to evaluate the diagnostic performance of AI models for the detection and quantification of OIRR. Overall, the findings indicate that deep learning systems—particularly CNN-based architectures—achieve excellent sensitivity and show strong agreement with manual reference standards, supporting their potential role in improving diagnostic workflows in orthodontic practice.

The pooled sensitivity across four eligible studies was 94%, indicating a consistently high ability to detect OIRR-positive cases. This aligns with the work of Xu et al. [23], who reported sensitivity of 0.97 using an EfficientNet-B1 architecture trained on CBCT slices, with reduced diagnostic variability and performance exceeding that of orthodontists. Similarly, Zheng et al. [17] developed a 3D model with sensitivity above 90%, supporting the pooled estimate and suggesting stable detection even in anatomically complex regions. Huang et al. [27] also reported substantial sensitivity, particularly in the apical third, where conventional approaches often underperform due to geometric distortion and limited contrast. Collectively, these findings suggest that CNN-based systems are not only accurate but also relatively robust to clinical variability, including artifacts and anatomical heterogeneity [31,32,33].

Specificity ranged from 82% to 98% across studies; however, heterogeneity in reporting formats and operational definitions prevented meta-analytic pooling. AUC values above 0.94 were reported by Zheng et al. [17], Huang et al. [27], and Xu et al. [26], demonstrating high discriminative capacity across different cohorts and resorption patterns. Xu et al. [25] reported similarly high AUC values in a multiclass grading framework, supported by Grad-CAM analyses highlighting the apical region as a decisive predictor. In contrast, Pirayesh et al. [30] reported comparatively lower performance (82% accuracy; F1-score 0.62), likely reflecting the limited training dataset and the focus on canine-induced resorption in a heterogeneous anatomical context. Prior methodological research has emphasized that constrained datasets can impair model generalizability and that data-efficient architectures and pre-training strategies may mitigate this limitation [34,35,36]. This consideration is consistent with the performance gap observed across studies in the present review, where differences in dataset size and design likely contributed to variability in diagnostic outcomes.

Agreement with manual reference standards was generally high. However, the pooled ICC of 1.000 should be interpreted cautiously. This near-perfect agreement may partly reflect methodological characteristics of the included studies, including the small number of studies contributing to the quantitative synthesis, limited external validation, and the use of highly standardized acquisition and annotation protocols. In addition, ICC estimates can be inflated by restricted variability in the assessed measurements (ceiling effect) or by shared operator-dependent reference standards, particularly when manual segmentation serves as ground truth under controlled conditions. Zheng et al. [17] and Lin et al. [28] reported excellent ICC values (>0.95) for volume-based measurements, suggesting that AI can reproduce clinically meaningful assessments in quantitative settings. Prior studies have also shown that AI-based measurements can reduce interobserver variability and dramatically decrease analysis time, further supporting clinical applicability [37,38]. Lin et al. [28] additionally reported that age, previous open bite, and deep overbite influenced resorption severity—factors not yet routinely incorporated into model development but potentially suitable for integration through multimodal learning or clinical feature embedding. Estrella et al. [29] reported more nuanced findings: while AI tracked root length changes closely, volumetric losses were often lower than those observed with manual assessments except in the apical third, where AI demonstrated greater precision. As previously noted, segmentation granularity and CBCT resolution can substantially influence the interpretation of apical morphology, reinforcing the need for optimization toward task-specific anatomical targets [39,40].

Exploratory subgroup patterns suggested potential sources of variability. Efficient Net and ensemble CNN-based approaches [25,28] tended to outperform more conventional architectures (e.g., ResNet-based or 3D U-Net models, such as those used by Pirayesh et al. [30]). Validation design also influenced reported accuracy: Estrella et al. [29] employed external multicenter validation and reported more conservative metrics, whereas most other studies relied on internal hold-out sets or cross-validation. It has been well established that internal validation can inflate performance estimates and limit real-world generalizability when overfitting is insufficiently controlled [41,42]. Accordingly, diagnostic metrics should be interpreted in light of validation strategy. The heterogeneity introduced by these methodological differences reinforces the need for harmonized AI reporting frameworks in orthodontic diagnostics.

Differences between volumetric and linear quantification approaches were also noteworthy. However, these comparisons are based on a limited number of studies and were not supported by formal subgroup meta-analysis; therefore, they should be regarded as exploratory and hypothesis-generating rather than confirmatory. Studies using volumetric assessments (Zheng et al. [17], Lin et al. [28], and Estrella et al. [29]) reported greater sensitivity for early resorptive changes and better characterization of regional variability. Estrella et al. [29] noted that apical 1–2 mm volumetric losses may be underestimated by manual segmentation but captured by AI, suggesting superior detection in narrow apical zones. Conversely, linear measurement approaches (Xu et al. [26] and Huang et al. [27]) emphasized ease of implementation and faster inference, but acknowledged potential underestimation of irregular lesions extending beyond the sagittal axis. This limitation has been recognized previously, and integration of volumetric mapping has been proposed where feasible [43,44]. Estrella et al. [29] further suggested that volumetric methods may be less sensitive to early apical remodeling due to dependence on complete tooth segmentation and accurate voxel calibration.

Overall risk of bias was low to moderate. Most studies reported clear eligibility criteria, blinded reference assessments, and adequate datasets; however, the limited use of external validation reduces generalizability and may lead to optimistic certainty estimates [17,26,27]. Despite these limitations, consistency in the direction of effects and the magnitude of improvement over conventional methods supports moderate to high certainty of evidence across the evaluated domains. Lin et al. [28] and Huang et al. [27] also emphasized that scalability requires prospective validation and integration of clinical variables, which aligns with the recommendations derived from this review. In addition to methodological heterogeneity (imaging protocols, architectures, and performance metrics), variability in outcome reporting precluded pooled estimates for several endpoints. Conversely, strengths of this review include a comprehensive search strategy, rigorous study selection, and inclusion of multiple high-quality studies using real patient CBCT data.

From a clinical perspective, integrating AI tools into OIRR detection workflows may improve diagnostic consistency, enable earlier intervention, and reduce the burden of manual segmentation. The ability of AI systems to replicate or exceed clinical-level performance, particularly in challenging regions such as the apical third, positions them as potentially valuable diagnostic aids. As highlighted by Zheng et al. [17], automated quantification may also facilitate individualized treatment planning and longitudinal monitoring. However, translation into routine practice will require robust external validation, software interoperability, and regulatory oversight.

Future research should focus on improving interpretability and explainability, incorporating multimodal clinical inputs (e.g., age, malocclusion type, and orthodontic force parameters), and conducting prospective multicenter studies with external test sets. Comparative evaluations of conventional 2D approaches versus AI-enhanced CBCT workflows should also address cost effectiveness and clinical impact. Furthermore, harmonization of performance reporting and data-sharing standards will be essential to translate high diagnostic potential into real-world orthodontic practice.

## 5. Conclusions

This systematic review and meta-analysis indicates that artificial intelligence models, particularly convolutional neural networks applied to CBCT imaging, demonstrate high diagnostic performance for detecting and quantifying orthodontically induced root resorption. The pooled sensitivity was 94%, with consistently high discriminatory capacity (AUC > 0.94) and strong agreement with manual reference standards (ICC > 0.95 in volumetric assessments), supporting the potential clinical value of AI-assisted CBCT interpretation in orthodontics. However, the substantial heterogeneity across studies limits the certainty and immediate generalizability of pooled estimates; therefore, clinical implementation should be considered cautiously until broader external validation becomes available. Future research should prioritize external validation across independent multicenter datasets and should adopt standardized reporting of AI model architectures and training pipelines (including preprocessing, segmentation strategy, model type, and evaluation metrics). Such standardization is essential to improve reproducibility and to reduce the substantial heterogeneity observed across studies.

## Figures and Tables

**Table 1:** (**A**) Study characteristics, CBCT parameters, and validation design of included studies; (**B**) AI model architectures, performance metrics, and efficiency/reproducibility notes.

(A)							
Study (Year), Country	Study Design	Sample Characteristics	CBCT Parameters	Target Region	Task Type	Reference Standard	Validation Design
Pirayesh et al. 2024 [30], Iran	Cross-sectional	CBCT scans of orthodontic patients (n = 80)	Voxel size: 0.2 mm; FOV: full arch	Full root	Segmentation + quantification	Manual volumetric segmentation	Train/test split (80/20)
Xu et al. 2024 [25], China	Cross-sectional	420 CBCT sagittal images (210 OIRR, 210 healthy)	Resolution: 0.125 mm; FOV: limited to anterior region	Apical third	Classification	Manual CBCT measurements	Train/test split (80/20)
Zheng et al. 2025 [17], China	Retrospective analysis	3000 tooth images from CBCT scans	FOV: 8 × 8 cm; Voxel size: 0.125 mm	Apical third	Segmentation + classification	Manual image classification	Fivefold cross-validation
Huang et al. 2025 [27], China	Diagnostic performance analysis	CBCT scans from 120 patients	Voxel size: 0.15 mm; FOV: posterior teeth	Entire root	Segmentation + classification	Manual segmentation	Cross-validation
Estrella et al. 2025 [29], Brazil	Prospective multicenter validation	CBCT data from 150 patients across 3 centers	Voxel size: 0.2 mm; FOV: full arch	Full root volume	Segmentation	Manual segmentation	External multicenter validation
Lin et al. 2025 [28], Taiwan	Quantitative diagnostic study	Pre- and post-treatment CBCT scans from 60 patients	Voxel size: 0.15 mm; FOV: full arch	Full root	Segmentation + quantification	Manual pre–post volume measurement	Internal validation
Xu et al. 2025 [26], China	Cross-sectional	CBCT images of anterior teeth (n = 250)	Voxel size: 0.125 mm	Root length (sagittal slice)	Classification	Manual measurement	Hold-out validation
(**B**)							
**Study**	**AI model architecture**	**Main performance metrics**		**Other metrics/notes**		**Efficiency/reproducibility**	
Pirayesh et al. [30]	3D U-Net	Dice = 0.93		Sensitivity = 0.91		Improved consistency vs. manual method	
Xu et al. [25]	EfficientNet-B1	Accuracy = 0.98		F1-score = 0.98		Outperformed orthodontists	
Zheng et al. [17]	Ensemble CNN architectures	Accuracy = 0.94; AUC = 0.96		—		Faster and more stable than manual	
Huang et al. [27]	Multi-scale CNN architecture	Dice = 0.91; AUC = 0.95		—		High automation and reproducibility	
Estrella et al. [29]	3D U-Net	Dice = 0.92		Sensitivity = 0.90		High generalizability and reproducibility	
Lin et al. [28]	Custom CNN	Volume accuracy = ±5%		Dice = 0.89		Reduced manual burden	
Xu et al. [26]	CNN	F1-score = 0.97; Accuracy = 0.95		—		Outperformed human raters	

AUC, area under the curve; CBCT, cone-beam computed tomography; CNN, convolutional neural network; Dice, Dice similarity coefficient; FOV, field of view; OIRR, orthodontically induced root resorption.

**Table 2:** Risk of bias assessment—QUADAS-2 (diagnostic accuracy studies).

Study	Patient Selection	Index Test	Reference Standard	Flow and Timing
Huang et al. [27]	Low	Low	Low	Low
Lin et al. [28]	Low	Low	Low	Low
Estrella et al. [29]	Low	Low	Low	Low

QUADAS-2 was applied to studies evaluating diagnostic accuracy. All three studies showed low risk of bias across domains.

**Table 3:** Risk of bias assessment—PROBAST (predictive modeling studies).

Study	Participants	Predictors	Outcome	Analysis
Zheng et al. [17]	Low	Low	Low	Moderate
Xu et al. [25]	Low	Low	Low	Moderate
Xu et al. [26]	Low	Low	Low	Moderate
Pirayesh et al. [30]	Low	Low	Low	Moderate

PROBAST was used for studies developing or validating predictive models. All studies demonstrated low risk in most domains, with moderate concerns related to the analysis domain.

**Table 4:** Summary of certainty of evidence (GRADE).

Outcome	Studies	Participants	Pooled Estimate	I^2^ (%)	Certainty	Downgrade Reasons
Sensitivity	5	~420	0.93 (0.89–0.96)	87.4%	Moderate	Imprecision, Heterogeneity
AUC	5	~420	Narrative	81.3%	Low	Inconsistency, Indirectness
ICC	3	~160	1.00	76.8%	High	None
Subgroup Analyses	2	~300	Narrative	74.6%	Low	Limited studies, Overlap of CIs

The certainty of evidence was rated using the GRADE approach for diagnostic test accuracy (DTA) studies [24]. Moderate certainty was assigned to pooled sensitivity due to heterogeneity and imprecision, while ICC outcomes showed high certainty. AUC and subgroup analyses received low certainty ratings due to inconsistency and limited generalizability.

## Data Availability

The original contributions presented in this study are included in the article and Supplementary Materials. Further inquiries can be directed to the corresponding author.

## Supplementary Materials

The following supporting information can be downloaded at: https://www.mdpi.com/article/10.3390/dj14020079/s1, Table S1: Database-specific search strategies and retrieved records. File S1: PRISMA 2020 checklist.
