# Supplementary material for: Artificial Intelligence Models for the Detection and Quantification of Orthodontically Induced Root Resorption Using Cone-Beam Computed Tomography: A Systematic Review and Meta-Analysis
Source: Dent J (Basel). 2026 Feb 2;14(2):79. doi: 10.3390/dj14020079 (PMC12939481; doi:10.3390/dj14020079)
Supplement: Supplementary file 1 [file dentistry-14-00079-s001.zip › dentistry-4112114-Supplementary Table S1.pdf]

Supplementary Table S1. Database-specific search strategies and retrieved records

Database	Search date (last search)	Search string (exact syntax)	Fields / controlled vocabulary	Limits	Records retrieved (n)
**PubMed/MEDLINE**	November 2025	((“Artificial Intelligence”[Mesh] OR “Machine Learning”[Mesh] OR “Deep Learning”[Mesh] OR “artificial intelligence”[tiab] OR “machine learning”[tiab] OR “deep learning”[tiab] OR CNN[tiab] OR “convolutional neural network*”[tiab]) AND (“Root Resorption”[Mesh] OR “root resorption”[tiab] OR “orthodontically induced root resorption”[tiab] OR OIRR[tiab] OR “external root resorption”[tiab]) AND (“Cone-Beam Computed Tomography”[Mesh] OR CBCT[tiab] OR “cone beam computed tomography”[tiab] OR “cone-beam computed tomography”[tiab]) AND (“Orthodontics”[Mesh] OR orthodontic*[tiab] OR “orthodontic treatment”[tiab] OR “orthodontic therapy”[tiab]))	MeSH + Title/Abstract	No language restrictions; no date restrictions	n = 587
**Scopus**	November 2025	TITLE-ABS-KEY(("root resorption" OR resorption OR "external root resorption" OR "external apical root resorption" OR "apical root resorption" OR OIRR OR EARR OR "orthodontically induced root resorption")	TITLE-ABS-KEY	No language restrictions; no date restrictions	n = 763

Database	Search date (last search)	Search string (exact syntax)	Fields / controlled vocabulary	Limits	Records retrieved (n)
		AND<br>(CBCT OR "cone beam" OR "cone-beam" OR<br>"cone beam computed tomography" OR "cone-beam computed tomography" OR<br>"cone beam CT" OR "cone-beam CT")<br>AND<br>("artificial intelligence" OR "machine learning" OR "deep learning" OR<br>"neural network*" OR CNN OR "convolutional neural network*" OR<br>"hierarchical deep learning" OR<br>automati* OR algorithm* OR "computer-aided" OR "computer assisted" OR<br>segmentation OR "image segmentation" OR "tooth segmentation" OR<br>quantif* OR grading OR detection OR diagnos*)<br>)			
**Web of Science Core Collection**	November 2025	TS=(<br>("root resorption" OR resorption OR<br>"external root resorption" OR "external apical root resorption" OR<br>"apical root resorption" OR<br>OIRR OR EARR OR<br>"orthodontically induced root resorption")<br>AND<br>(CBCT OR "cone beam" OR "cone-beam" OR<br>"cone beam computed tomography" OR "cone-	Topic (TS)	No language restrictions; no date restrictions	n = 289

Database	Search date (last search)	Search string (exact syntax)	Fields / controlled vocabulary	Limits	Records retrieved (n)
		beam computed tomography" OR<br>"cone beam CT" OR "cone-beam CT")<br>AND<br>("artificial intelligence" OR "machine learning" OR "deep learning" OR<br>"neural network*" OR CNN OR "convolutional neural network*" OR<br>"hierarchical deep learning" OR<br>automati* OR algorithm* OR "computer-aided" OR "computer assisted" OR<br>segmentation OR "image segmentation" OR "tooth segmentation" OR<br>quantif* OR grading OR detection OR diagnos*)<br>)			
**Embase (Elsevier)**	November 2025	(<br>'root resorption'/exp OR<br>'root resorption':ti,ab,kw OR<br>'external root resorption':ti,ab,kw OR<br>'external apical root resorption':ti,ab,kw OR<br>'apical root resorption':ti,ab,kw OR<br>oirr:ti,ab,kw OR<br>earr:ti,ab,kw OR<br>'orthodontically induced root resorption':ti,ab,kw<br>)<br>AND<br>(<br>'cone beam computed tomography'/exp OR<br>cbct:ti,ab,kw OR<br>'cone beam':ti,ab,kw OR<br>'cone-beam':ti,ab,kw OR	Emtree + ti,ab,kw	No language restrictions; no date restrictions	n = 472

Database	Search date (last search)	Search string (exact syntax)	Fields / controlled vocabulary	Limits	Records retrieved (n)
		'cone beam computed tomography':ti,ab,kw OR<br>'cone-beam computed tomography':ti,ab,kw OR<br>'cone beam ct':ti,ab,kw OR<br>'cone-beam ct':ti,ab,kw<br>)<br>AND<br>(<br>'artificial intelligence'/exp OR<br>'machine learning'/exp OR<br>'deep learning'/exp OR<br>'neural network'/exp OR<br>'convolutional neural network'/exp OR<br>'image segmentation'/exp OR<br>'artificial intelligence':ti,ab,kw OR<br>'machine learning':ti,ab,kw OR<br>'deep learning':ti,ab,kw OR<br>'neural network*':ti,ab,kw OR<br>cnn:ti,ab,kw OR<br>'convolutional neural network*':ti,ab,kw OR<br>'hierarchical deep learning':ti,ab,kw OR<br>automati*:ti,ab,kw OR<br>algorithm*:ti,ab,kw OR<br>'computer-aided':ti,ab,kw OR<br>'computer assisted':ti,ab,kw OR<br>segmentation:ti,ab,kw OR<br>'image segmentation':ti,ab,kw OR<br>'tooth segmentation':ti,ab,kw OR<br>quantif*:ti,ab,kw OR<br>grading:ti,ab,kw OR			

Database	Search date (last search)	Search string (exact syntax)	Fields / controlled vocabulary	Limits	Records retrieved (n)
		detection:ti,ab,kw OR<br>diagnos*:ti,ab,kw<br>)<br>AND<br>(<br>orthodont*:ti,ab,kw OR<br>'orthodontics'/exp OR<br>'orthodontic treatment':ti,ab,kw<br>)			
